# Evidence for inhibition of cholinesterases in insect and mammalian nervous systems by the insect repellent deet

**DOI:** 10.1186/1741-7007-7-47

**Published:** 2009-08-05

**Authors:** Vincent Corbel, Maria Stankiewicz, Cédric Pennetier, Didier Fournier, Jure Stojan, Emmanuelle Girard, Mitko Dimitrov, Jordi Molgó, Jean-Marc Hougard, Bruno Lapied

**Affiliations:** 1 Laboratoire de Lutte contre les Insectes Nuisibles, Institut de Recherche pour le Développement, F-34 394 Montpellier, France; 2 Institute of General & Molecular Biology, N. Copernicus University, 87-100 Torun, Poland; 3 Institut de Recherche pour le Développement/Centre de Recherches Entomologiques de Cotonou, 01 BP 4414, République du Bénin; 4 Laboratoire Récepteurs et Canaux Ioniques Membranaires (RCIM), UPRES EA 2647/USC INRA 2023, IFR 149 QUASAV, Université d'Angers, UFR Sciences, F-49045 Angers cedex, France; 5 Groupe de Biotechnologie des protéines, Université de Toulouse, Toulouse, France; 6 Institute of Biochemistry, Medical Faculty, University of Ljubljana, Slovenia; 7 CNRS, Institut de Neurobiologie Alfred Fessard – FRC2118, Laboratoire de Neurobiologie Cellulaire et Moléculaire – UPR9040, Gif sur Yvette, F-91198, France

## Abstract

**Background:**

N,N-Diethyl-3-methylbenzamide (deet) remains the gold standard for insect repellents. About 200 million people use it every year and over 8 billion doses have been applied over the past 50 years. Despite the widespread and increased interest in the use of deet in public health programmes, controversies remain concerning both the identification of its target sites at the olfactory system and its mechanism of toxicity in insects, mammals and humans. Here, we investigated the molecular target site for deet and the consequences of its interactions with carbamate insecticides on the cholinergic system.

**Results:**

By using toxicological, biochemical and electrophysiological techniques, we show that deet is not simply a behaviour-modifying chemical but that it also inhibits cholinesterase activity, in both insect and mammalian neuronal preparations. Deet is commonly used in combination with insecticides and we show that deet has the capacity to strengthen the toxicity of carbamates, a class of insecticides known to block acetylcholinesterase.

**Conclusion:**

These findings question the safety of deet, particularly in combination with other chemicals, and they highlight the importance of a multidisciplinary approach to the development of safer insect repellents for use in public health.

## Background

The use of repellents against biting arthropods was probably developed a thousand years ago [1]; however, a real breakthrough occurred in 1953 with the discovery of the synthetic repellent N,N-Diethyl-3-methylbenzamide (deet), which became the most commonly used active ingredient of topically applied insect repellent due to its efficacy against a broad spectrum of medically important pests, including mosquitoes [2]. Despite the widespread and increased interest in the use of deet in public health programmes [3-5], controversies remain concerning both the identification of its target sites at the molecular level and its exact mechanism of action in insects. Ditzen and colleagues [6] suggested that deet may block electrophysiological responses of olfactory sensory neurons to attractive odours in *Anopheles gambiae *Giles (Diptera:Culicidae) and *Drosophila melanogaster *Meigen (Diptera:Drosophilidae). By contrast, Syed and Leal [7] have recently reported that mosquitoes detect deet by means of olfaction, a physiological mechanism that directly initiates avoidance behaviour (i.e., deet does not cause a loss of attractive chemical signal).

Although the debate concerning the 'olfactory' mode of action of deet is still a topical question, other laboratory bioassays and field experiments have revealed that deet also exerts a deterrent effect in insects and has insecticidal properties [8-10]. In the same context, if deet is considered to have a relatively good toxicological profile [11], other authors have shown that excessive doses of deet could be toxic to humans and could cause severe seizures and lethality when combined with other active ingredients, such as pesticides [12-14]. It has been reported previously that symptoms related to deet poisoning in invertebrates, mammals and humans reflect an apparent action on the central nervous system (CNS) [15-18]. Based on these findings, we have investigated further the potential mechanisms of deet toxicity. For the first time, we have identified a molecular target site for deet (i.e., cholinesterases) in both insect and mammal neuronal preparations, and have investigated the consequences of its interactions with carbamate insecticides on the cholinergic system.

## Results and discussion

### Insecticidal effect of *deet *on insects

**Figure 1:**
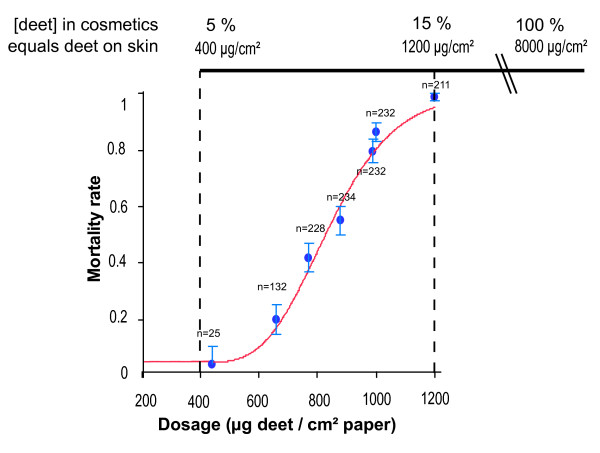
**Insecticidal effect of deet on mosquitoes**. **a) **Mortality rate among *A. aegypti *exposed for 1 h to paper impregnated with deet in World Health Organisation bioassays. Doses applied on paper were compared with standard skin applications of commercially available formulations containing deet (lower and upper deet formulation concentrations 5 to 100% were taken from the review of Xue et al. [20]). Deet formulation concentrations (%) were converted to doses (μg/cm^2^) based on 5 ml being the average volume required to cover a human arm [4]. The figure showed that the doses used on skin (400 μg/cm^2 ^to 8000 μg/cm^2^) were equivalent or greater than doses showing insecticidal properties in the case of close contact (LD_50 _= 830 ± 30 μg/cm^2^, s.e.m; LD_95 _= 1,180 ± 50 μg/cm^2^).

To elucidate repellent toxicity in insects, we first assessed the sensitivity of the dengue vector *Aedes aegypti *L. (Diptera:Culicidae) to deet -treated filter papers using World Health Organization (WHO) bioassays [19]. Figure 1 shows that deet caused dose-dependent mortality at doses ranging from 400 to 1200 μg/cm^2 ^[4,20]. This range corresponds to the lower range of doses usually applied to human skin for personal protection. Topical applications of deet on the mosquito *Culex pipiens quinquefasciatus *Say (Diptera:Culicidae) resulted in an LD_50 _(lethal dose for 50% of exposed mosquitoes) and an LD_90 _of deet for adult females of 393.3 ± 25.4 (standard error of the mean; s.e.m.) and 1103.0 ± 25.4 ng of active ingredient/mg of mosquito (ng a.i./mg, respectively). For comparison, the LD_50 _and LD_90 _of propoxur (2-(1-Methylethoxy) phenol methylcarbamate) were 2.6 ± 0.2 and 10.5 ± 1.6 ng a.i./mg female, respectively, indicating that the amount of deet required to kill mosquitoes was about 150 times higher than that for propoxur, an acetylcholinesterase (AChE, EC 3.1.1.7) inhibitor. However, the slopes of the regression lines for mosquito mortality for deet (3.67 ± 0.85) and propoxur (3.35 ± 0.50) did not differ significantly. This indicated a similar heterogeneity of response by the mosquitoes with respect to the toxic effect of the two molecules.

### Neurophysiological effects of deet on insect and mammalian neuronal preparations

**Figure 2:**
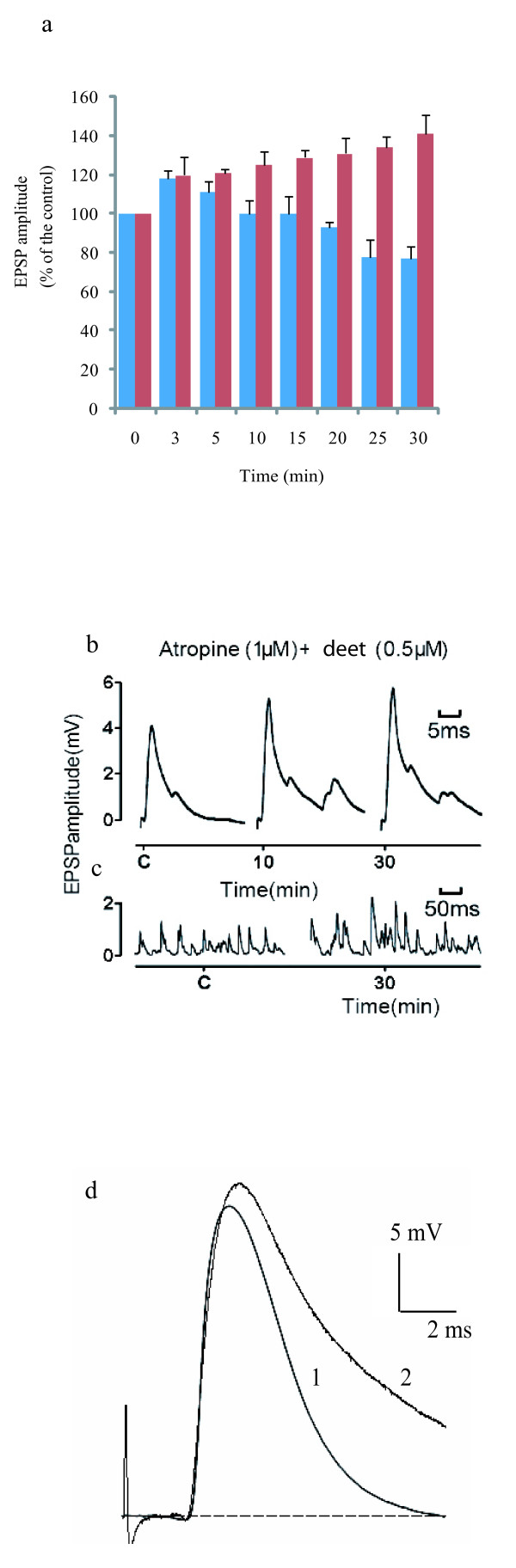
**Effects of deet on insect and mammalian neuronal preparations**. **a) **The Histogram illustrates the excitatory postsynaptic potential (EPSP) amplitudes (% of control) versus time (min) after exposure to 1 μM of deet in absence (blue bars) and in the presence of atropine (red bars) in *P. americana *central nervous system. Application of deet without atropine (blue bars) induced a biphasic effect on EPSP amplitudes. Within the first 3 min, application of deet induced a significant increase of EPSP amplitude which reflected an elevation of acetylcholine (ACh) concentration in the synaptic cleft (see text for details). After 3 min, a significant EPSP depression was observed, suggesting a regulation of ACh concentration in the synaptic cleft through an activation of presynaptic muscarinic receptors. Pre-treatment for 10 min with atropine 1 μM (red bars) clearly reversed the EPSP depression observed with deet 1 μM, confirming the participation of the muscarinic receptors in the negative feedback of ACh release following deet exposure. Data are means ± S.E.M. **b) and c**) Typical examples of cockroach composite (b) and unitary (c) EPSP following deet application. Experiments were done in the presence of atropine (1 μm) to prevent an action of presynaptic muscarinic receptors. Note the increase of unitary EPSP frequency and amplitude (c) following deet application (0.5 μM) in the synapses. **d**) Effect of deet on the time course of full size endplate potentials (EPPs) recorded in a mouse hemidiaphragm preparation bathed with a standard Krebs-Ringer solution supplemented with 1.6 μM μ-conotoxin GIIIB to selectively block sodium channels in muscle fibres. The EPPs recorded under control conditions (trace 1), and after the addition of 500 μM deet to the medium (trace 2); note the prolongation of the decay phase of EPPs in the presence of deet, with little change in the amplitude and time to peak; the mean decay-time constant were 11.1 ± 0.7 and 3.8 ± 0.08 ms for deet-treated and controls, respectively (*n *= 6, *P *< 0.001).

Based on these observations, we investigated the neurophysiological effects of deet on the cercal-afferent giant-interneuron synapses in the terminal abdominal ganglion of the cockroach *Periplaneta americana *L. (Dyctioptera: Blattidae), known to present many functional analogies with other insect systems [21]. The single-fibre oil-gap method [22] is a well-adapted electrophysiological technique for the cockroach CNS, which allows investigation of the effects of such compounds at the synaptic level. Deet dissolved in physiological saline was applied at two concentrations (0.5 and 1 μM) on the synaptic preparation by superfusion into the experimental chamber. Bath application of deet produced a biphasic effect on excitatory post synaptic potential (EPSP) amplitudes. As illustrated in Figure 2a (blue bars), deet (1 μM) produced an increase in EPSP amplitude within the first 3 min (118 ± 4% s.e.m., *F*_1.18 _= 29, *P *< 0.001, *n *= 10, Figure 2a). This effect was also observed at 0.5 μM deet (113 ± 5% s.e.m. at 0.5 μM, *F*_1.14 _= 32, *P *< 0.001, *n *= 8). After 3 min, a time-dependent decrease in EPSP amplitude was observed compared with controls, which was more pronounced with the higher concentration (77 ± 6% after 30 min, *F*_1.12 _= 26, *P *< 0.001, *n *= 6, Figure 2a). This typical biphasic effect, previously observed with anticholinesterase compounds such as carbamates [23], reflected changes in synaptic transmission activity. Indeed, treatment with carbamates can cause an increase in acetylcholine (ACh) concentration that is sufficient to activate negative feedback acting through presynaptic muscarinic receptors, which thereby decrease subsequent release of ACh [23,24]. As the deet-induced biphasic effect on EPSP amplitude was very similar to that reported with classical anticholinesterase compounds, it is possible that deet might cause an elevation of ACh concentration into the synaptic cleft via an inhibition of AChE. To test this hypothesis, we conducted additional experiments in the presence of atropine, known to block muscarinic receptors in insect synaptic transmission [24]. As illustrated in Figure 2a (red bars), pre-treatment with atropine (1 μM) for 10 min counteracted the EPSP depression previously observed with deet, producing only a time-dependent increase in EPSP amplitude (for example, 142 ± 4% after 30 min exposure to 1 μM deet, *F*_1.18 _= 27, *P *< 0.001, *n *= 10). These data confirm the participation of pre-synaptic muscarinic receptors in the modulation of ACh release in the synapses after bath application of deet [24]. It is also interesting to note that application of deet on synaptic preparations, pre-treated with 1 μM atropine, increased both composite (Figure 2b) and unitary EPSP amplitudes (Figure 2c), which result from the spontaneous activity of presynaptic cercal mechanoreceptors. All these findings clearly indicate that ACh is not efficiently hydrolyzed by AChE in the presence of deet.

Based on these unexpected results and because AChE is an ubiquitous enzyme in both insect and mammalian nervous systems, additional electrophysiological studies were performed on isolated mouse phrenic hemidiaphragm muscles. We showed that 500 μM deet prolonged by about threefold the decay time constant of synaptic potentials on endplate regions of the muscle fibre (Figure 2d). This prolongation of the time course of synaptic potentials, which is known to occur after AChE inhibition [25,26] or in the absence of AChE expression [27], was shown to be due to the lack of ACh hydrolysis, allowing ACh to persist in the synaptic cleft and to activate endplate nicotinic ACh receptors repeatedly. Considering our data, higher concentrations of deet were, however, required to prolong the decay time constant of synaptic events on mammalian neuromuscular preparations (500 μM) compared with cockroach synaptic preparations (1 μM).

### Characterization of cholinesterase inhibition by deet

**Figure 3:**
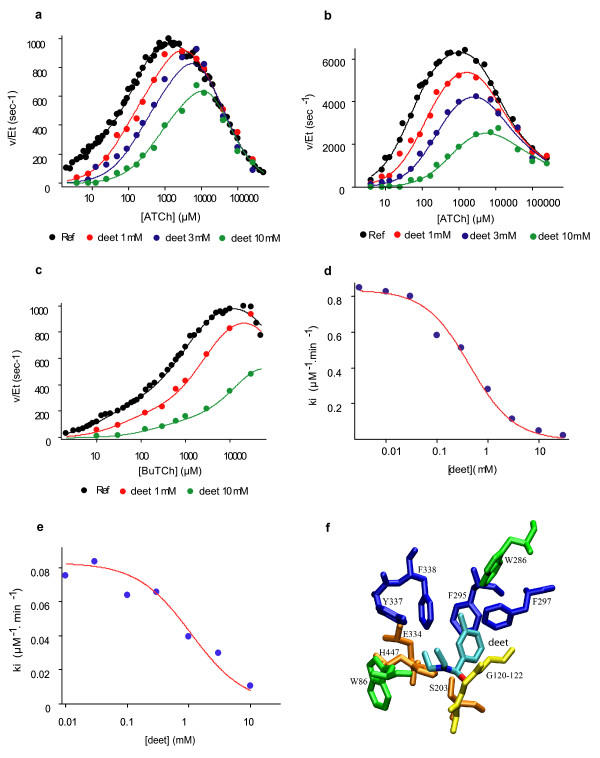
**Effects of deet on cholinesterase enzymatic activities**. **a) and b) **Inhibition of *D. melanogaster *(a) and Human (b) acetylcholinesterases (AChEs) by deet. Note the dose-dependant decrease of ATCh hydrolysis by AChE following deet application. [ATCh]: Acetylthiocholine concentration in micromole per liter; v/[Et] specific activity in s-1. **c) **Inhibition of human (Hu) butyrylcholinesterase by deet. As previously observed with ATCh, deet is also capable of strongly decreasing the BTCh hydrolysis by human BChE. [BTCh]: butyrylthiocholine concentration in micromole per liter; v/[Et] specific activity in s-1. **d and e) **Dose-dependant effect of deet on *Drosophila *(d) and Human (e) AChE carbamoylation rates by propoxur (carbamate). The curves clearly show the strong reduction of the second order rate constant (ki) for the carbamoylation of HuAChE by propoxur in presence of deet. At high concentration (10 mM), protection of AChE by deet is total. **f) **Accommodation and binding of deet inside the active site of Human AChE. The picture was created by VMD, a Visual Molecular Dynamics program. After QMMM relaxation of the complex between HuAChE and deet molecule, the latter was accommodated in a tetrahedral adduct conformation. Minimal adaptation of the side chains of adjacent residues in the active side of HuAChE suggests that the accommodation of deet in a position favourable for enzymatic hydrolysis is possible.

To ascertain the inhibition of cholinesterases by deet, we analysed, *in vitro*, the effect of deet on the activity of purified AChE from *D. melanogaster *(DmAChE) and both acetyl and butyrylcholinesterases (EC 3.1.1.8) from human (HuAChE and HuBChE). As illustrated in Figures 3a, b, and 3c, incubation of each enzyme with the substrate and deet (from 1 to 10 mM) resulted in a strong reduction of enzyme activity. This indicates that deet is capable of inhibiting the hydrolysis of acetylthiocholine (ATCh) and butyrylthiocholine (BTCh) by AChEs. As preincubation of the enzyme with deet in the absence of substrate did not change the extent of inhibition, and as dilution of the inhibited enzyme restored enzyme activity, Deet can be considered as a reversible inhibitor of cholinesterases. Deet has also the capacity to diminish the rate of AChE carbamoylation by propoxur (Figures 3d and 3e), indicating that both molecules act as competitive inhibitors for the enzyme.

The kinetics of substrate hydrolysis by cholinesterases are complex. The substrate first binds to a peripheral site, located at the entrance of the active site gorge, and then slides down to the catalytic site, buried 20 Å inside the protein (Additional file 1). Simultaneous kinetic analyses of inhibition of substrate hydrolysis and carbamoylation allowed us to estimate the binding constants of DEET for the two substrate binding sites of cholinesterases. Binding of deet at the peripheral site was estimated to be 1.02 ± 0.03, 8.39 ± 6.97 and 0.37 ± 0.05 mM for DmAChE, HuAChE and HuBChE, respectively (Table 1). Binding of deet at the catalytic site located at the bottom of the active site gorge was not necessary to describe inhibition of DmAChE and was estimated as 4.67 and 1.08 mM for HuAChE and HuBChE, respectively (Table 1). Thus, deet would enter into the active site gorge of HuAChE and HuBChE, but not that of DmAChE, resulting in a stronger inhibition of human enzymes. This hypothesis was consistent with structural data showing the active site gorge of DmAChE to be about 50% narrower than the active site of HuAChE [28]. To determine whether the accommodation and binding of deet was possible within the active site of vertebrate AChE, it was docked as a tetrahedral adduct on the catalytic serine of human AChE crystal structure (1B41). Minimal adaptation of the side chains of adjacent residues in the active side of HuAChE suggests the accommodation of deet in a catalytic site is possible (Figure 3f).

**Table 1:** Characteristic kinetic constants for the hydrolysis of ATCh and BTCh by DmAChE, HuAChE and HuBChE

	***DmAChE (ATCh)***	***HuAChE (ATCh)***	***HuBChE (BTCh)***
**Substrate**			
*k*_3 _[s^-1^]	395.4	13471 ± 2171	1082 ± 44
*K*_*ip *_[*m*M]	0.17	5.43 ± 1.0	11.3 ± 0.5
*K*_*L*_	4.17	0.0181 ± 0.0054	0.0019 ± 0.0008
*K*_*LL*_	179	9.23 ± 6.58	12.3 ± 5.4
*k*_2 _[s^-1^]	53465	14375 ± 2182	420 ± 20
*A*	3.44	1.64 ± 0.36	1.32 ± 0.07
*B*	0.049	0.11 ± 0.03	39.6 ± 13.5
*K*_*s *_= *K*_p_*K_*L *_[*m*M]	0.73	0.098	0.021
*K*_*ss *_= *K*_p_*K_*LL *_[*m*M]	31.1	50.1	139

**DEET**			
*K*_*ip *_[*m*M]	1.02 ± 0.03	8.39 ± 6.97	0.37 ± 0.05
*K*_*iL*_		0.083 ± 0.078	1.54 ± 0.45
*K*_*iLL*_		0.56 ± -0.41	29 ± 5.4
*c*		1.49 ± 0.99	1.32
*d*		0.19 ± 0.18	0.52 ± 0.09
*K*_*is *_= *K*_ip_*K_*iL *_[*m*M]		0.69	0.57
*K*_*iss *_= *K*_ip_*K_*iLL *_[*m*M]		4.67	1.08

ATCh = acetylthiocholine; BTCh = butyrylthiocholine; DmAChE = Acetylcholinesterase from *D. melanogaster*; HuAChE = Acetylcholinesterase from human; HuBChE = Butyrylcholinesterase from human.

### Interactions between deet and cholinesterase compounds

**Figure 4:**
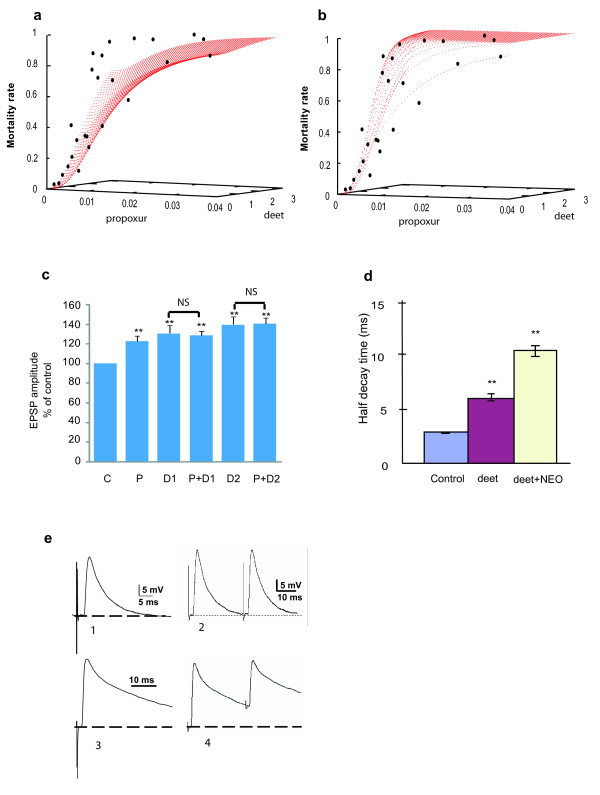
**Interactions between deet and anti-cholinesterasic compounds**. **a) and b) **Toxic interactions between deet (μg/mg mosquito) and propoxur (μg/mg mosquito) for *C. quinquefasciatus *by topical application. The model including a synergistic interaction (b) between the two molecules provided a better description of the data than a model based on simply additive effects (a); see equations  where *ED *is the effective dose, *D *the dose and *ic *the interaction coefficient. The interaction coefficient (*ic *= 3.07 ± 0.98) was significantly greater than 0, indicating that deet synergised propoxur toxicity in insects. **c) **Effects of propoxur (P) and deet (D), alone and in combination (P+D), on cockroach synaptic activity. All synaptic preparations were pretreated (10 min) with atropine (1 μM). NS not significant (*P *> 0.05). **d) **Effect of deet and neostigmine on the time course of full size EPPS recorded in mouse hemidiaphragm preparations. Mean values (± s.e.m, *n *= 6) of the half-decay time of EPPs (ms) under control conditions (2.8 ± 0.05 ms, blue column), 500 μM deet (6.1 ± 0.36 ms, red column) and in the continuous presence of deet and 3 μM neostigmine (10.5 ± 0.55 ms, yellow column). * denotes a significant difference from controls (*P *< 0.001). **e) **Examples of full size endplate potentials (EPPs) in response to a single or paired stimulus in the presence of 500 μM deet and in the presence of deet (upper part) and 3 μM neostigmine (lower part). * denote significant difference from control *P *< 0.001.

Having established that deet binds to the active site of cholinesterases and then hinders the entrance of substrates, we investigated its potential interaction with carbamate insecticides. The effects of topical applications of a range of deet doses combined with a range of propoxur doses applied to *C. quinquefasciatus *were not in agreement with a model based on the hypothesis of an additive effect for the two compounds (Figure 4a). Several models of interactions between the two chemicals were subsequently tested. The best fit took into account a synergistic interaction involving the effect of deet on the insecticidal effect of propoxur (Figure 4b). Further electrophysiological experiments were conducted on *P. americana *preparations to assess deet and propoxur interactions at the synaptic level (Figure 4c). After pre-treatment of atropine and when applied alone, both propoxur (P) and deet (D1 and D2) significantly increased EPSP amplitude compared with the control (*P *< 0.01 and *P *< 0.001, respectively). In the presence of atropine, however, subsequent application of deet + propoxur (P+D) did not cause a greater effect on post-synaptic potentials than that caused by deet alone at the same concentrations. Propoxur+D1 was almost equal to D1; the difference between them was not significant (*F*_1.16 _= 0, *P *< 0.95, *n *= 8). Similarly, there was no difference between P+D2 and D2 (*F*_1.16 _= 0, *P *< 0.92, *n *= 8). This indicates that propoxur and deet acted similarly on the same target site in the insect cholinergic system.

However, a different trend was noted on mouse isolated phrenic hemidiaphragm muscles. Indeed, when neostigmine (3 μM) was perfused in the continuous presence of 500 μM deet, the decay time constant of synaptic responses was about two fold more prolonged than with deet alone (Figure 4d). Recordings of full (maximum) size endplate potentials (EPPs) in response to a single or paired nerve stimuli, either in the presence of 500 μM deet or in the presence of deet plus 3 μM neostigmine, showed a marked prolongation of the decay phase of the EPPs in the presence of deet and neostigmine (Figure 4e). These results indicated that deet (i) had an inhibitory action on AChE in mouse hemidiaphragm endplates that was not maximal at the concentration used; (ii) did not prevent subsequent action of neostigmine on endplate AChE; and (iii) was less active, on an equimolar basis, than neostigmine in mouse hemidiaphragm junctions. We have shown that deet causes an equal or even greater *in vitro *inhibitory effect on purified human enzyme than on insect AChE, and therefore speculate that deet may be a less potent inhibitor of the asymmetric forms of AChEs, which are anchored to the basal lamina of the mouse skeletal neuromuscular junction[29];

## Conclusion

*In vivo *toxic interactions between deet and propoxur, pirimiphos-methyl, or pyridostigmine bromide (PB) for cockroaches and mosquitoes have been reported previously [8,30,31]. In adult hens, Abou-Donia *et al*. [18] demonstrated that co-exposure to sub-neurotoxic doses of PB, deet and chlorpyrifos resulted in increased toxicity characterized by neurological dysfunction and neuropathological lesions. In the central cholinergic system of rats, application of physiologically relevant doses of pyridostigmine and deet, in combination, led to neurobehavioural deficits and region-specific alterations in AChE and nicotinic receptors [32]. More investigations are urgently needed to confirm or dismiss the potential neurotoxicity to humans arising from the combined use of deet with different cholinesterase inhibitors.

## Methods

### Toxicological studies

#### Biological materials

The standard insecticide susceptible strains 'S-Lab' of *C. quinquefasciatus *and 'Bora' of *A. aegypti *were used in bioassays. These two strains have been colonized for many years at IRD-LIN in Montpellier and are free of any detectable insecticide resistance mechanisms.

#### Topical applications

Topical solutions were first prepared by dissolving technical grade deet 97% (Sigma-Aldrich, Saint Quentin Fallavier, France) and/or propoxur 99.6% (Bayer CropScience, Monheim, Germany) in acetone. For each compound, five to eight doses were used to provide a range of mortality from 0 to 100%. Two-to-five-day-old non-blood-fed females of *C. quinquefasciatus *were first anaesthetised by extended contact with carbon dioxide then placed on a refrigerated plate at 4°C to maintain anaesthesia during manipulation [19]. A volume of 0.1 μl of insecticide solution (at the required concentration) was deposited on the upper part of the pronotum of females using a micro-capillary. Females receiving a volume of 0.1 μl pure acetone served as controls. After each test, females were transferred into plastic cups and provided with 10% honey solution on cotton wool and held for 24 h at 27°C and 80% relative humidity. Mortality rates were recorded 24 h after the tests. Data were analysed with the program Global Optimization by Simulated Annealing (GOSA) [33]using the statistical approach according to Finney [34]. Mortality (*y*) as a function of deet doses (*x*) was fitted to the cumulative Gauss function and was expressed in nanograms of insecticide per milligram of female weight.



#### Treated filter papers bioassay

Mortality resulting from tarsal contact with treated filter paper was measured using WHO test kits [19] against adult females of *A. aegypti*. Four batches of 25 non-blood-fed females, two to five days old, were introduced into WHO bioassay holding tubes for a period of 60 min. They were then transferred to exposure tubes, which were held vertically for 60 min under subdued light. Mortality was recorded 24 h after exposure. Each solution was tested four times and each test was replicated three times with different cohorts of insects to take into account inter-batch variability.

### Electrophysiology

#### Insect preparations

Adult male cockroaches *P. americana *were taken from our laboratory stock colonies which are maintained under standard conditions (29°C photo-cycle 12 h light/12 h dark). Cockroaches were pinned dorsal side up in a dissection dish and dorsal cuticles were removed to allow access to the ventral nerve cord. The terminal abdominal ganglion (TAG) with the nerve cord were carefully dissected and placed in normal cockroach saline containing (in mM): NaCl 208, KCl 3.1, CaCl_2 _10, sucrose 26, 4-(2-hydroxyethyl)-1-piperazineethanesulfonic acid (HEPES) 10; pH was adjusted to 7.2 with NaOH. The synaptic preparation was composed of a cercus, the corresponding cercal nerve XI, the de-sheathed TAG (containing the studied synapse) and the abdominal part of the nerve cord. Electrophysiological recordings of synaptic events were obtained using the single-fibre oil-gap method [22]. With this technique it is possible to record unitary excitatory postsynaptic potentials (uEPSP) resulting from the activity of pre-synaptic cercal mechanoreceptors and composite EPSP. These potentials were triggered in response to short electrical pre-synaptic stimulation applied at a frequency of 0.1 Hz to the ipsilateral cercal nerve XI and are the main subject of observations to study synaptic transmission. During experiments, the resting potential was continuously monitored on a pen chart recorder. The uEPSPs and EPSPs were recorded using a Hameg oscilloscope and stored on a PC computer with Hameg software. Experiments were conducted at room temperature (20°C). Data were expressed as a mean ± s.e.m. when quantified. Electrophysiological data were analysed for statistical significance using a one-way Analysis of Variance (ANOVA) followed by a post-hoc Tukey test. Differences among data were judged to be significant when *P *< 0.05. Data analysis was performed using STATISTICA (StatSoft, Cracow, Poland).

In all electrophysiological experiments, deet and propoxur were prepared in dimethylsulfoxide (DMSO, stock solution 10 mM) and absolute ethanol (stock solution 10 mM), respectively. Final dilutions in physiological saline contained at most 0.1% DMSO and absolute ethanol. These concentrations of solvents had no effect on synaptic transmission. All compounds used were purchased from Sigma Chemicals (L'isle d'Abeau Chesnes, France) and propoxur was bought from Bayer AG (Leverkusen, Germany).

#### Mammalian preparations

All experiments on mice were performed in accordance with French and European Community guidelines for laboratory animal handling [35]. Adult male Swiss-Webster mice (20 to 25 g body weight) purchased from IFFA CREDO (Saint Germain sur l'Arbresle, France) were anaesthetized with Isoflurane (AErrane^®^, Baxter S.A., Lessines, Belgium) inhalation, and euthanized by dislocation of the cervical vertebrae followed by immediate exsanguination. The left mouse hemidiaphragm with its associated phrenic nerve was dissected out from the animal and mounted in a silicone-lined organ bath (4 ml volume). Isolated preparations were perfused with standard Krebs-Ringer solution of the following composition: 154.0 mM NaCl, 5.0 mM KCl, 2 mM CaCl_2_, 1.0 mM MgCl_2_, 5.0 mM HEPES, and 11.0 mM glucose. The solution gassed with pure O_2 _had a pH of 7.4.

Electrophysiological recordings on isolated phrenic hemidiaphragm muscles were performed using conventional techniques [27]. Briefly, membrane and synaptic potentials were recorded from endplate regions, at room temperature (22°C), with intracellular microelectrodes filled with 3 M KCl (8–12 MΩ resistance), or with extracellular microelectrodes (filled with Krebs-Ringer solution, 1–3 MΩ resistance) and an Axoclamp-2A system (Axon Instruments, Foster City, CA, USA). Electrical signals after amplification were displayed on a digital oscilloscope, collected and digitized at a sampling rate of 25 kHz with the aid of a PC computer and a Digidata 1322A unit (Axon Instruments). Computerized data acquisition and analysis was performed with the program WinWCP (V3.8), provided by Dr John Dempster (University of Strathclyde, Strathclyde, Scotland). The motor nerve of isolated neuromuscular preparations was stimulated via a suction microelectrode, adapted to the diameter of the nerve, with square wave pulses of 0.1 ms duration, generated by a S-44 stimulator (Grass Instruments, AstroMed, W. Warwick, RI, USA), and supramaximal intensity (typically 3–8 V). Studies on EPPs were performed in standard physiological solution containing 1.6 μM μ-conotoxin *Conus Geographus *(GIIIB) (Alomone Labs, Jerusalem, Israel) to block voltage-dependent sodium channels of skeletal muscle fibres [36]. The amplitudes of full-sized EPPs and MEPPs recorded on junctions treated with μ-conotoxin GIIIB were normalized to a membrane potential of -75 mV. MEPPs and EPPs were analysed individually for amplitude and time course. For each condition studied, four to six individual experiments were performed and the results were averaged to give the presented mean ± s.e.m. The statistical significance of differences between controls and test values was assessed with Student's *t*-Test (two-tailed), or the Kolmogorov-Smirnov two-sample test. Differences were considered significant if *P *< 0.05.

### Biochemistry

DmAChE was produced in the baculovirus system and purified as previously described [37]. The native human AChE and BChE used for kinetic studies were from Sigma Chemical Co. (St Louis, MO, USA). Hydrolysis of ATCh was measured spectrophotometrically at 412 nm by the Ellman method [38] at 25°C, in 25 mM phosphate buffer, pH 7. Substrate concentrations were 4 μM-200 mM, with a minimum of five repetitions per concentration. Activity was followed for 1 min after addition of the enzyme to the mixture and spontaneous hydrolysis of the substrate was subtracted. Rates of carbamoylation were estimated by incubation of AChEs with various concentrations of propoxur for different periods of time. The remaining activity was measured for 30 sec following 10-fold dilutions in Ellman reaction medium supplemented with 1 mM acetylthiocholine. Data were analysed using the model and equation of Stojan and colleagues [39] for ATCh hydrolysis inhibition and using the model of pseudo first order irreversible inhibition for carbamoylation rate. Fits were performed simultaneously on both equations by multiple non-linear regressions using the program GOSA [33].

### Molecular docking of deet into AChE

The accommodation and binding of deet inside the active site of HuAChE was made by building a 3D structure of deet using MOLDEN, a processing program of molecular and electronic structure, and then optimized quantum mechanically *in vacuo *by Gaussian 03, an electronic structure program. For the calculation we used 6–31 g* basis set at the Hartree-Fock level. For molecular mechanics, energy and dynamic calculations we assigned atomic types for the deet molecule already existing in the CHARMM distribution C27n1 topology file. Charges were calculated by Mullikan's approximation and the missing parameters were searched until a satisfactory fit of the model to the *ab initio *energy potentials and geometry was obtained.

In the next step we manually docked the deet in the active site above the catalytic serine (S203) of the HuAChE molecule: the appropriate three atoms of deet were superimposed on the corresponding atoms of substrate analogue molecule situated in the active site of torpedo AChE (PDB code 2C5F) with the carbonyl oxygen pointing into the oxyanion hole. The structure was then fully relaxed without moving any of the protein atoms. Finally, our 3D model of HuAChE and docked deet was subjected to two successive 50-step QMMM refinements, assigning the deet molecule, catalytic serine (S203) and histidine (H447) quantum mechanically (49 QM atoms and two link atoms), while the rest of protein and water molecules (193 of them) were treated mechanically. During QMMM relaxation of the complex between HuAChE and the deet molecule, the latter was accommodated in a tetrahedral adduct conformation.

## Competing interests

The authors declare that they have no competing interests.

## Authors' contributions

VC, CP, DF and BL. designed the experiments and wrote the paper. JMH and CP conducted the toxicological experiments on mosquitoes. MS performed the electrophysiological studies on cockroaches, and DF and MD carried out the biochemical studies on insect and human purified cholinesterases. JS modelled the binding of deet into the active site of human AChE, and EG and JM conducted the electrophysiological experiments on mouse neuromuscular junctions. All authors discussed the results and contributed to the text and statistical analyses. All authors have read and approved the final manuscript.

## Supplementary Material

Additional file 1 Reaction scheme for substrate hydrolysis by cholinesterases in the presence of a reversible inhibitor that competes at the peripheral anionic and the catalytic site of the free and acetylated enzyme. Click here for file

## References

[B1] Moore SJ, Debboun M, Debboun M, Frances SP, Strickman D (2006). The history of insect repellents. INSECT REPELLENTS, Principles, Methods, and Uses.

[B2] Fradin MS (1998). Mosquitoes and mosquito repellents: a clinician's guide. Ann Intern Med.

[B3] Rowland M, Downey G, Rab A, Freeman T, Mohammad N, Rehman H, Durrani N, Reyburn H, Curtis C, Lines J (2004). DEET mosquito repellent provides personal protection against malaria: a household randomized trial in an Afghan refugee camp in Pakistan. Trop Med Int Health.

[B4] Costantini C, Badolo A, Ilboudo-Sanogo E (2004). Field evaluation of the efficacy and persistence of insect repellents DEET, IR3535, and KBR 3023 against Anopheles gambiae complex and other Afrotropical vector mosquitoes. Trans R Soc Trop Med Hyg.

[B5] Durrheim DN, Govere JM (2002). Malaria outbreak control in an African village by community application of 'deet' mosquito repellent to ankles and feet. Medical and Veterinary Entomology.

[B6] Ditzen M, Pellegrino M, Vosshall LB (2008). Insect odorant receptors are molecular targets of the insect repellent DEET. Science.

[B7] Syed Z, Leal WS (2008). Mosquitoes smell and avoid the insect repellent DEET. Proc Natl Acad Sci USA.

[B8] Moss JI (1996). Synergism of toxicity of N,N Diethyl m toluamide to German Cockroaches (Ortoptera: Blattelidae) by Hydrolytic Enzyme Inhibitiors. Journal of Economical Entomology.

[B9] Licciardi S, Herve JP, Darriet F, Hougard JM, Corbel V (2006). Lethal and behavioural effects of three synthetic repellents (DEET, IR3535 and KBR 3023) on Aedes aegypti mosquitoes in laboratory assays. Med Vet Entomol.

[B10] N'Guessan R, Rowland M, Moumouni TL, Kesse NB, Carnevale P (2006). Evaluation of synthetic repellents on mosquito nets in experimental huts against insecticide-resistant Anopheles gambiae and Culex quinquefasciatus mosquitoes. Trans R Soc Trop Med Hyg.

[B11] Koren G, Matsui D, Bailey B (2003). DEET-based insect repellents: safety implications for children and pregnant and lactating women. Cmaj.

[B12] Clem JR, Havemann DF, Raebel MA (1993). Insect repellent (N,N-diethyl-m-toluamide) cardiovascular toxicity in an adult. Ann Pharmacother.

[B13] Lipscomb JW, Kramer JE, Leikin JB (1992). Seizure following brief exposure to the insect repellent N,N-diethyl-m-toluamide. Ann Emerg Med.

[B14] Schaefer C, Peters PW (1992). Intrauterine diethyltoluamide exposure and fetal outcome. Reprod Toxicol.

[B15] Abou-Donia MB, Dechkovskaia AM, Goldstein LB, Abdel-Rahman A, Bullman SL, Khan WA (2004). Co-exposure to pyridostigmine bromide, DEET, and/or permethrin causes sensorimotor deficit and alterations in brain acetylcholinesterase activity. Pharmacol Biochem Behav.

[B16] Abdel-Rahman A, Abou-Donia S, El-Masry E, Shetty A, Abou-Donia M (2004). Stress and combined exposure to low doses of pyridostigmine bromide, DEET, and permethrin produce neurochemical and neuropathological alterations in cerebral cortex, hippocampus, and cerebellum. J Toxicol Environ Health A.

[B17] Chaney LA, Rockhold RW, Mozingo JR, Hume AS, Moss JI (1997). Potentiation of pyridostigmine bromide toxicity in mice by selected adrenergic agents and caffeine. Vet Hum Toxicol.

[B18] Abou-Donia MB, Wilmarth KR, Abdel-Rahman AA, Jensen KF, Oehme FW, Kurt TL (1996). Increased neurotoxicity following concurrent exposure to pyridostigmine bromide, DEET, and chlorpyrifos. Fundam Appl Toxicol.

[B19] WHO, ed (2006). Guidelines for testing mosquito adulticides intended for Indoor Residual Spraying (IRS) and Insecticide Treated Nets (ITNs).

[B20] Xue RD, Ali A, Day JF, Debboun M, Frances SP, Strickman D (2007). Commercially Available Insect Reppellents and Criteria for Their Use. Insect Reppellents: Principles, Methodes and Uses.

[B21] Matsumura F (1985). Toxicology of insecticides.

[B22] Hue B, Callec JJ, Huber I, Masler EP, Rao BR (1990). Electrophysiology and pharmacology of synaptic transmission in central nervous system of the cochroach. Cockroaches as models for neurobiology: applications in biochemical research.

[B23] Corbel V, Stankiewicz M, Bonnet J, Grolleau F, Hougard JM, Lapied B (2006). Synergism between insecticides permethrin and propoxur occurs through activation of presynaptic muscarinic negative feedback of acetylcholine release in the insect central nervous system. Neurotoxicology.

[B24] Hue B, Lapied B, Malecot C (1989). Do presynaptic muscarinic receptors regulate acetylcholine release in the central nervous system of the cockroach Periplaneta americana?. J Exp Biol.

[B25] Katz B, Miledi R (1973). The binding of acetylcholine to receptors and its removal from the synaptic cleft. J Physiol.

[B26] Kloot W Van der, Balezina OP, Molgo J, Naves LA (1994). The timing of channel opening during miniature endplate currents at the frog and mouse neuromuscular junctions: effects of fasciculin-2, other anti-cholinesterases and vesamicol. Pflugers Arch.

[B27] Minic J, Chatonnet A, Krejci E, Molgo J (2003). Butyrylcholinesterase and acetylcholinesterase activity and quantal transmitter release at normal and acetylcholinesterase knockout mouse neuromuscular junctions. Br J Pharmacol.

[B28] Harel M, Kryger G, Rosenberry TL, Mallender WD, Lewis T, Fletcher RJ, Guss JM, Silman I, Sussman JL (2000). Three-dimensional structures of Drosophila melanogaster acetylcholinesterase and of its complexes with two potent inhibitors. Protein Sci.

[B29] Feng G, Krejci E, Molgo J, Cunningham JM, Massoulie J, Sanes JR (1999). Genetic analysis of collagen Q: roles in acetylcholinesterase and butyrylcholinesterase assembly and in synaptic structure and function. J Cell Biol.

[B30] Pennetier C, Corbel V, Hougard JM (2005). Combination of a non-pyrethroid insecticide and a repellent: a new approach for controlling knockdown-resistant mosquitoes. Am J Trop Med Hyg.

[B31] Pennetier C, Corbel V, Boko P, Odjo A, N'Guessan R, Lapied B, Hougard JM (2007). Synergy between repellents and non-pyrethroid insecticides strongly extends the efficacy of treated nets against Anopheles gambiae. Malar J.

[B32] Abu-Qare AW, Abou-Donia MB (2001). Simultaneous determination of malathion, permethrin, DEET (N,N-diethyl-m-toluamide), and their metabolites in rat plasma and urine using high performance liquid chromatography. J Pharm Biomed Anal.

[B33] Global Optimization by Simulated Annealing (GOSA). Global Optimization by Simulated Annealing (GOSA).. http://www.bio-log.biz.

[B34] Finney D (1971). Probit Analysis.

[B35] French and European Community guidelines for laboratory animal handling. http://ec.europa.eu/environment/chemicals/lab_animals/proposal_en.htm.

[B36] Montero-Solis C, Gonzalez-Ceron L, Rodriguez MH, Cirerol BE, Zamudio F, Possanni LD, James AA, de la Cruz Hernandez-Hernandez F (2004). Identification and characterization of gp65, a salivary-gland-specific molecule expressed in the malaria vector Anopheles albimanus. Insect Mol Biol.

[B37] Chaabihi H, Fournier D, Fedon Y, Bossy JP, Ravallec M, Devauchelle G, Cerutti M (1994). Biochemical characterization of Drosophila melanogaster acetylcholinesterase expressed by recombinant baculoviruses. Biochem Biophys Res Commun.

[B38] Ellman GL, Courtney KD, Andres V, Feather-Stone RM (1961). A new and rapid colorimetric determination of acetylcholinesterase activity. Biochem Pharmacol.

[B39] Stojan J, Golicnik M, Fournier D (2004). Rational polynomial equation as an unbiased approach for the kinetic studies of Drosophila melanogaster acetylcholinesterase reaction mechanism. Biochim Biophys Acta.

