# Correction: Evidence for inhibition of cholinesterases in insect and mammalian nervous systems by the insect repellent deet

**DOI:** 10.1186/1741-7007-10-86

**Published:** 2012-10-31

**Authors:** Vincent Corbel, Maria Stankiewicz, Cédric Pennetier, Didier Fournier, Jure Stojan, Emmanuelle Girard, Mitko Dimitrov, Jordi Molgó, Jean-Marc Hougard, Bruno Lapied

**Affiliations:** 1 Laboratoire de Lutte contre les Insectes Nuisibles, Institut de Recherche pour le Développement, F-34 394 Montpellier, France; 2 Institute of General & Molecular Biology, N. Copernicus University, 87-100 Torun, Poland; 3 Institut de Recherche pour le Développement/Centre de Recherches Entomologiques de Cotonou, 01 BP 4414, République du Bénin; 4 Laboratoire Récepteurs et Canaux Ioniques Membranaires (RCIM), UPRES EA 2647/USC INRA 2023, IFR 149 QUASAV, Université d'Angers, UFR Sciences, F-49045 Angers cedex, France; 5 Groupe de Biotechnologie des protéines, Université de Toulouse, Toulouse, France; 6 Institute of Biochemistry, Medical Faculty, University of Ljubljana, Slovenia; 7 CNRS, Institut de Neurobiologie Alfred Fessard - FRC2118, Laboratoire de Neurobiologie Cellulaire et Moléculaire - UPR9040, Gif sur Yvette, F-91198, France

## Correction

After the publication of this work [1] it was brought to our attention that there were insufficient details given on the final composition of the experimental and control samples used in some experiments in this study. We would like to clarify that the statement made in the methods, "Final dilutions in physiological saline contained at most 0.1% DMSO and absolute ethanol" applies to the electrophysiological experiments done on insect preparations, but not to those on mammalian preparations. Note that 0.1% DMSO has no effect on insect synaptic transmission. We supply all previously missing details in this correction.

For the experiment recording endplate potentials in a mouse hemidiaphragm preparation (Figure two d), in which 500 μM deet was applied, the final concentration of DMSO was 5% in both the sample treated with deet and the control (traces 1 and 2, Figure two d). For the experiment looking at the interaction between DEET and the insecticide neostigmine on a mouse hemidiaphragm preparation (Figure four d and e), the concentration of DMSO was 5% in both control samples and those bathed in 500 μM deet.

For the experiment looking at the effects of deet and propoxur on cockroach synaptic activity (Figure four c), deet and propoxur were prepared in dimethylsulfoxide (DMSO, stock solution 10 mM) and absolute ethanol (stock solution 10 mM), respectively, and diluted in normal cockroach saline to give 0.5 μM deet (D1), 1 μM deet (D2) and 0.2 μM propoxur (P) in the experimental samples, whereas control samples were maintained in normal cockroach saline alone. Final dilutions in physiological saline contained at most 0.1% DMSO and absolute ethanol. These concentrations of solvents had no effect on synaptic transmission.

We would also like to clarify that for the biochemical experiments in Figure three, the deet was diluted from a 2 M stock solution in absolute ethanol, so that for the maximum concentration of deet used (20 mM, Figure three d), the concentration of ethanol was 1%. Control samples did not contain ethanol, but we have found in other experiments that 1% ethanol has no effect on the kinetics of several acetylcholinesterases (see [2]).

## References

[B1] Corbel V Stankiewicz M Pennetier C Fournier D Stojan J Girard E Dimitrov M Molgo J Hougard JM Lapied B Evidence for inhibition of cholinesterases in insect and mammalian nervous systems by the insect repellent deet BMC Biol 2009 7 47 10.1186/1741-7007-7-47 19656357 PMC2739159

[B2] Fekonja O Zorec-Karlovsek M El Kharbili M Fournier D Stojan J Inhibition and protection of cholinesterases by methanol and ethanol J Enzyme Inhib Med Chem 2007 22 4 407 415 10.1080/14756360601143857 17847706

