# Supplementary material for: Evidence for inhibition of cholinesterases in insect and mammalian nervous systems by the insect repellent deet
Source: BMC Biol. 2009 Aug 5;7:47. doi: 10.1186/1741-7007-7-47 (PMC2739159; doi:10.1186/1741-7007-7-47)
Supplement: Additional file 1 — Reaction scheme for substrate hydrolysis by cholinesterases in the presence of a reversible inhibitor that competes at the peripheral anionic and the catalytic site of the free and acetylated enzyme. [file 1741-7007-7-47-S1.doc]

**Additional files**

**Additional data 1:** Reaction scheme for substrate hydrolysis by cholinesterases in the presence of a reversible inhibitor that competes at the peripheral anionic and the catalytic site of the free and acetylated enzyme.


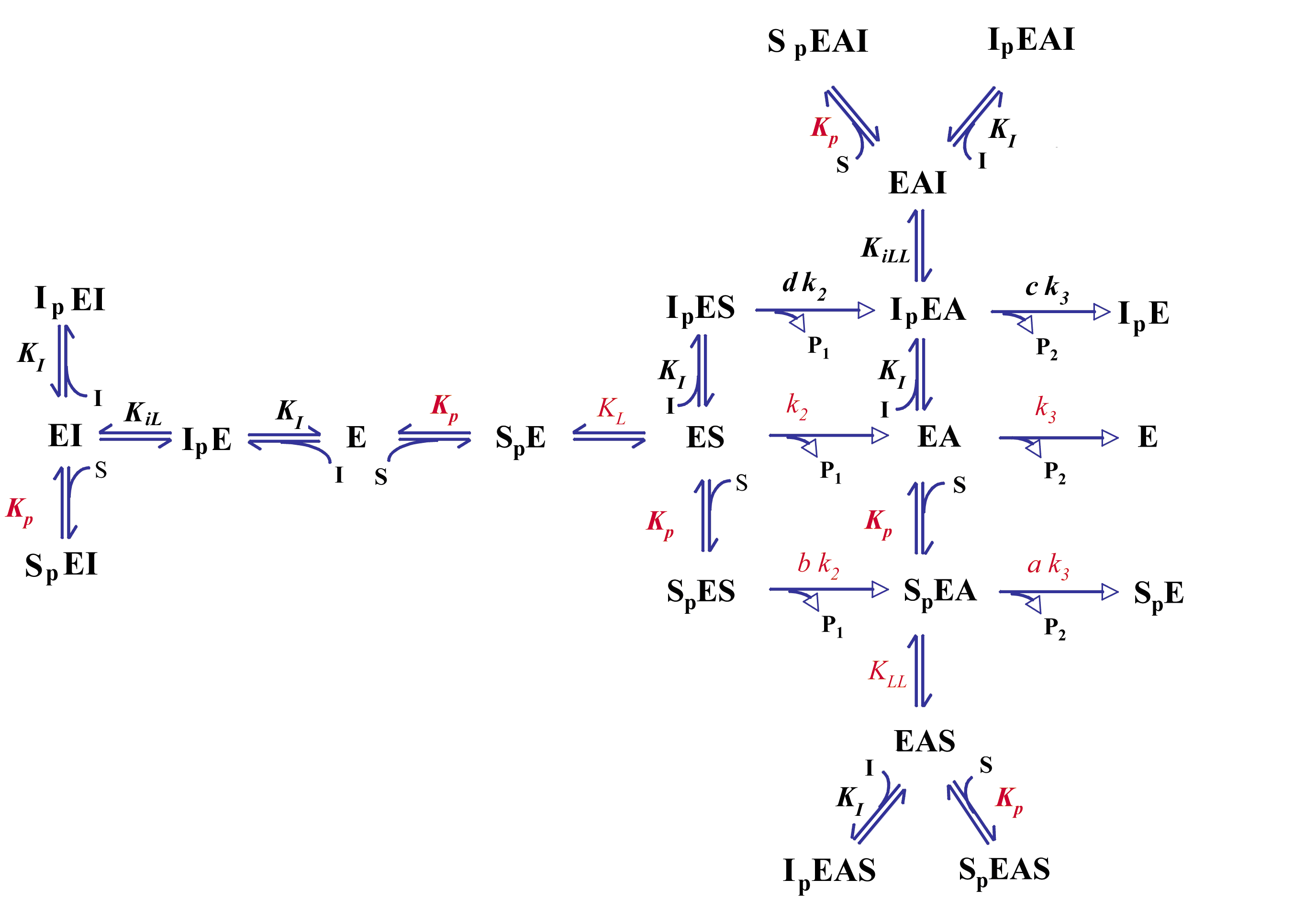


E: free enzyme and its legacy, S: substrate, I: inhibitor, SpE, IpE: substrate or inhibitor bound to the peripheral anionic site, ES, EI: substrate or inhibitor bound to the catalytic site, EA: acetylated enzyme and its legacy. In this model, the substrate initially binds to a peripheral site located at the rim of the active site (SpE, *Kp*) and then slides down to the bottom (ES, *K_L_*) where it is hydrolyzed. The enzyme is acetylated (EA, *k_2_*) and the first product, choline (P_1_), is released by the gorge entrance. Then deacetylation occurs (*k_3_*) regenerating the free enzyme. The existence of two binding subsites allows the second substrate molecule to attach to the peripheral site before (SpES) and during the catalysis at the acylation site (SpEA). Moreover, when the enzyme gets acylated and the choline is released, the two substrate molecules can fully occupy and thus block the active site of cholinesterases (SpEAS, see structures with PDB codes 2C4H and 2HA4 for Torpedo and mouse AChE).
